# Classification of coronoid process fractures: A pending question

**DOI:** 10.3389/fsurg.2022.890744

**Published:** 2022-08-02

**Authors:** Daofeng Wang, Jiantao Li, Gaoxiang Xu, Wupeng Zhang, Li Li, Peifu Tang, Licheng Zhang

**Affiliations:** ^1^ Department of Orthopedics, The Fourth Medical Center of Chinese PLA General Hospital, Beijing, China; ^2^ National Clinical Research Center for Orthopedics, Sports Medicine and Rehabilitation, Beijing, China; ^3^ School of Medicine, Nankai University, Tianjin, China

**Keywords:** coronoid fracture, classification, combined injuries, injury pattern, treatment

## Abstract

Ulna coronoid fracture is a complicated elbow injury. Comprehensive classification of coronoid fracture can assist diagnosis, guide treatment, and improve prognosis. Existing coronoid fracture classifications are insufficient to interpret all fracture patterns. The coronoid fracture classification is associated with elbow-specific trauma patterns. Coronoid fractures are often associated with other elbow injuries, commonly with radial head fractures, which makes the clinical strategies inconsistent and prognosis poor. The current fracture classifications do not contain information about combined injuries. Preservation of ulnohumeral joint contact after trauma is critical to elbow mechanical and kinematic stability. Important fracture types for treatment include terrible-triad injuries and anteromedial facet fractures. Open reduction and internal fixation of these two fractures should be conducted when marked displacement of the fragment, elbow instability under stress, and complicated associated injuries. The current surgical tactics based on classifications are still controversial.

## Introduction

The coronoid process was recognized as the most important bone stabilizer for elbow stability (1). Together with the radial head, the coronoid process is involved in the formation of the anterior osseous buttress of the great sigmoid fossa, preventing backward displacement of the elbow (2). In addition, the coronoid process also provides important soft-tissue attachment sites including the anterior bundle of the ulnar collateral ligament, the brachialis muscle and the anterior capsule, and maintains elbow stability with these structures.

Nearly 58% of the anteromedial facet was unsupported by the proximal ulnar metaphysis and diaphysis, making it vulnerable during varus and posteromedial rotation violence (3). Coronoid fractures occur in about 2%–10% of complex elbow fractures and dislocations, often accompanied by other important structures injuries, such as radial head and ligament complex (4, 5), making treatment principles ambiguous. The coronoid fracture classifications have greatly standardized the clinical treatment. However, with the comprehension of fracture features and the evolution of the treatment concept, the existing fracture classifications cannot cover all fracture types. For decades, many studies have interpreted the classifications, concomitant injuries and management of coronoid fracture, but there are still several differences in each part and no consensus has been reached.

## Classification: Proof and evaluation

### Coronoid height

**Figure 1:**
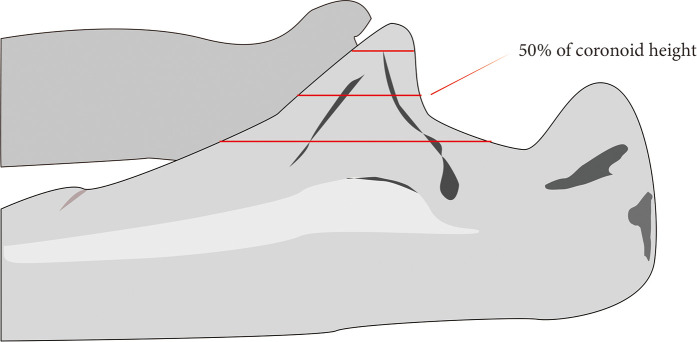
Regan-Morrey classification. Type 1, avulsion of the coronoid process tip; type 2, fractures involving less than 50% of the coronoid process; type 3, fractures involving more than 50% of the coronoid process.

Morrey (4) firstly proposed the fracture classification based on the coronoid height on the lateral radiograph (Figure 1) and further analyzed the posterior displacement of the elbow from flexion to extension after removing different volumes of coronoid height and found the 50% coronoid height was necessary for elbow stability (1, 6). They called 50% coronoid height the “minimal required articulation”. Since then, numerous morphologic studies have described coronoid height, but there are still no unified conclusions on the definition, measurement methods and results of coronoid height at present (7).

Morrey defined a line drawn parallel to the ulnar shaft through the olecranon tip, it bisects approximately 50% of the coronoid (8). Doornberg pointed out that the coronoid base should be a line connecting the base of the trochlear notch and the anterior ulnar cortical margin distal to the coronoid (3, 9). Matzon proposed 4 identifiable points to define coronoid bases: the trough of trochlear notch, the transverse groove of trochlear notch at the guiding ridge, the anterior ulna distal to coronoid where the slope changes, and the anterior ulna at distal brachialis insertion (10). Furthermore, the literature description of the coronoid base is not limited to those mentioned above.

The controversies on coronoid height also exist between Regan and Morrey (R-M) type 1 and type 2. Ring (2, 9) noted that the definition of coronoid height between R-M 1,2 types was vague, and there was a contradiction in the definition of classification combined with other injuries. Subsequently, Doornberg (11) defined the fractures involving the joint capsule as type 2 due to the insertion site of the joint capsule being about 5 mm away from the coronoid tip (12), while the injuries only involving tip as type 1. They also pointed out that coronoid height in terrible-triad injuries (TTIs) varied greatly (19%–59%, average 35%) and could not be simply divided by R-M classification. Fracture classification based on fracture morphology and injury mechanism may be more reliable (9).

### Fracture patterns

**Figure 2:**
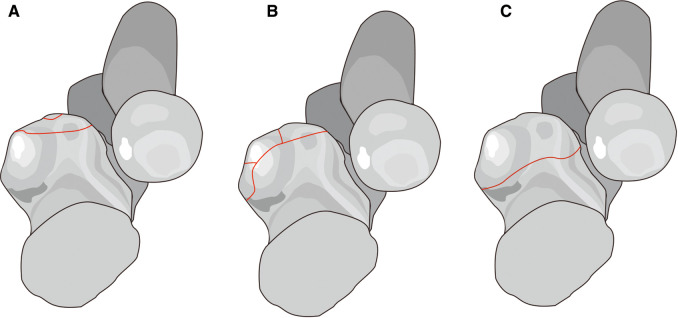
O’Driscoll classification. Type 1, coronoid tip fracture (**A**); type 2, anteromedial rim plus tip fracture (**B**); type 3, anteromedial rim and sublime tubercle fractures with or without the involvement of the tip fracture (**C**).

After the R-M classification was introduced, researchers discovered the fracture features were more than just transverse or comminuted fractures as described in the first classification. In 2003, O'Driscoll proposed a new classification based on the injury pattern (Figure 2) that stressed anteromedial facet fractures (AMFs) and deepened the understanding of the injury mechanism (12). Subsequently, Sanchez-Sotelo (13) reported two oblique compression fractures on the anteromedial facet caused by varus and posteromedial rotation instability injury. This fracture was relatively hidden and often accompanied by elbow instability and sublocation, revealing the O'Driscoll classification was more comprehensive.

In addition, Mellema (14) analyzed 110 coronoid fracture heat mapping based on 3DCT and found the morphology of coronoid fracture was in line with the O'Driscoll classification. However, studies have also found the anterolateral fracture independent of the O'Driscoll classification. This type belongs to the coronoid tip fracture in TTIs and the fracture lines were relatively lateral. Adams (15) found the coronoid injury patterns should also include oblique fractures on the coronoid anterolateral facet (7%), and called for further studies to verify the feasibility of this new type. Recently, Rhyou (16) also found that the patients developing coronoid fractures combined with radial head injuries often involved the coronoid anterolateral facet. This further suggested the existing fracture classifications were insufficient to cover all fracture patterns.

## Classification and injury

### Injury patterns

Injury patterns of traumatic elbow instability are correlated with coronoid fracture classification. The correlation analysis between coronoid fracture and elbow trauma showed that R-M type2 was closely associated with TTIs and varus posteromedial rotational instability (VPMRI) patterns, while R-M type1 did not correlate with elbow injury patterns (11, 14). A biomechanical study pointed out the posterolateral rotatory injury mechanism (PLRI) can easily lead to small coronoid fractures, namely R-M type1–2 and O'Driscoll type1 fractures (17). In contrast, VPMRI pattern can result in O'Driscoll type 2 fractures: the fractured anteromedial facet with the disrupted lateral collateral ligament (12, 13, 18, 19). Type3 fractures almost involved the olecranon disorders with elbow dislocation caused by axial force (11, 14).

Coronoid fractures were often combined with other elbow injuries, making treatment complicated and inconsistent (5, 11, 20) (Table 1). It is very important for surgeons to accurately understand the correlations during fracture management. Previous studies focused on how to maximize the rehabilitation and maintenance of elbow stability after fracture, but there was little analysis on the relationship between fracture types and concomitant injuries. Morrey (4) noticed that the associated injuries were the major determinant of treatment and the combination with other lesions can lead to prolonged fixation time and increase the risk of serious complications such as heterotopic ossification. Recently, Adams (5) followed up 103 fractures and found that the patients with other elbow injuries had lower MEPS, smaller extension and rotation range, and more obvious elbow pain. However, the study did not expound on the correlation between classification and comorbidities.

**Table 1:** The association between injury patterns, mechanism and coronoid fracture classifications and combined injuries of elbow.

Combined injuries	IPs	Mechanism	R-M type			O’Driscoll type				
			1	2	3	1	2-1	2–2	2–3	3
Radial head	TT	PLRI		+		+				+
Olecranon	OFD	Axial violence			+					+
LCL	VPMRI + PLRI	VPMRI + PLRI		+			+	+	+	
MCL	VPMRI	VPMRI		+		+	+	+	+	
Elbow subluxation	VPMRI + PLRI	VPMRI + PLRI		+				+	+	
Elbow dislocation	All above	All above		+	+	+	+			+

IPs, injury patterns; R-M type, Regan-Morrey classification; LCL, lateral collateral ligament complex; MCL, medial collateral ligament complex; PLRI, posterolateral rotatory injury mechanism; TT, terrible-triad fracture-dislocation; OFD, olecranon fracture–dislocation; VPMRI, varus posteromedial rotational instability pattern fracture–dislocation.

### Radial head fracture involvement

**Figure 3:**
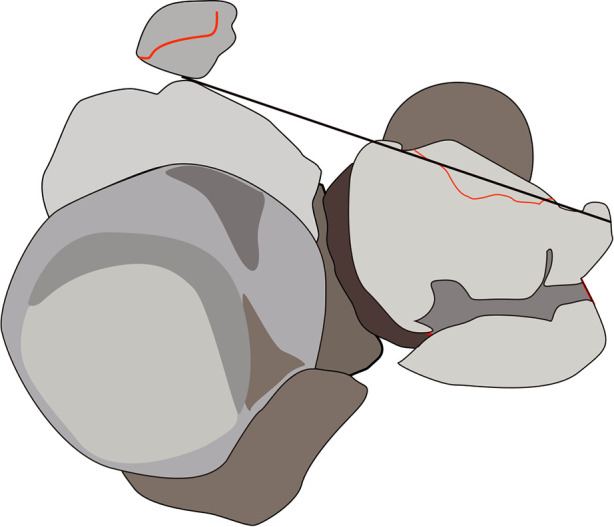
Associated injuries. Illustration demonstrates the fracture line (Solid black line) of coronoid process and radial head comminuted fractures.

Radial head injuries are the most common type of combined comorbidities (4, 18) (Figure 3). Most studies believed that R-M type1 fracture only includes coronoid tip and does not involve other intraarticular osseous structure and soft tissue injuries (11, 18, 21). Ring found that R-M type2 injuries often combine with radial head fracture and elbow subluxation. Additionally, they also noted that R-M type 2 was associated with elbow VPMRI pattern, suggesting AMF fracture was common in R-M type 2 (11).

Although the specific subtype was not specified, previous studies believed that the O'Driscoll type 1 involving anterior joint capsule insertion was associated with radial head fracture (11, 18). Heat mapping of coronoid fracture showed that the fracture in posterior olecranon fracture-dislocation (POFD) mainly involved the apex, and was significantly associated with O'Driscoll type1 and TTIs, as well as posterior Monteggia fracture-dislocation (14). A radiographic analysis on TTIs discovered that the most common combinations of coronoid fractures associated with radial head injuries were M2R2 (Mason type2 combined with R-M type2) and M2O1 (Mason type2 combined with O'Driscoll type1) (22). Furthermore, several studies proposed a new coronoid anterolateral fracture and detected the clinical relevance between that and radial head fractures (14–16).

### Olecranon fracture association

Coronoid fracture with olecranon fracture-dislocation was caused by axial violence, which often reduces the elbow ROM after surgery and requires additional surgical intervention (5). Doornberg (11) found that 22 of 24 patients with olecranon fracture-dislocation had large coronoid fracture fragments indicating that O'Driscoll type 3 and R-M type 3 were often associated with olecranon fractures. Subsequently, Adams observed nearly half of the patients with coronoid basic fractures were complicated with transverse olecranon fractures according to the fracture lines characteristics (15). Mellema (14) analyzed the coronoid fracture line distribution based on 3DCT and found that the fracture line exited at the coronoid base in olecranon fracture-dislocation patients, further confirming the correlation between type 3 and olecranon fracture. Besides these, few studies reported olecranon injury in certain AMF fractures, namely O'Driscoll type 2 fractures (23, 20).

### Elbow dislocation combined

Morrey found the elbow joint has a natural tendency for posterior displacement based on the study of osseous constraints maintaining the elbow stability (1), explaining the high incidence of elbow posterior dislocation in clinical settings. The elbow dislocation was common in TTIs (2, 11, 14) while subluxation was common in AMFs (11). The specific type of elbow dislocation in AMF fracture was related to the fragment size: AMFs with small fragments were usually seen in type 2-1, and were accompanied by elbow dislocation, otherwise, the elbow subluxation. Park (24) concluded that O'Driscoll type 2-1 was associated with elbow posterior dislocation, while type 2-2, 2–3 were combined with varus subluxation in AMFs. This pattern discrepancy may be due to the different injury mechanisms between fracture subtypes: type 2-1 fracture are mainly caused by rotational force, while type 2-2, 2–3 fractures are more prone to shearing force. Most studies have shown that R-M type 3 and O'Driscoll type 3 basal fractures are usually larger fracture fragments and often associated with olecranon fracture-dislocation (5, 11, 14, 25).

### Lateral collateral igament complex

Since O'Driscoll (12) proposed AMF fracture caused by VPMRI injury, there are increasing studies that have paid attention to this pattern due to its difficulty in early detection and significant effect on elbow function. Sanchez-Sotelo (13) reported two coronoid medial oblique compression fractures, which featured the positive posterolateral rotatory drawer and lateral pivot shift tests, indicating the deficiency of the LCL. Weber (18) pointed out that O'Driscoll type 2 was combined with LCL injuries, while medial collateral ligament (MCL) injuries were relatively rare. Doornberg found that 15 of 18 AMF fractures with LCL injuries were complicated with elbow varus subluxation after follow-up analysis (23). Rhyou (16) respectively analyzed the ligament injuries in coronoid fractures alone, and combined injuries and found that simple coronoid injury often occurred on the AMF and the risk of LCL rupture was greater (OR = 3.5). In combined injuries, the incidence of ligament injury was greater (10/15; 66.7%). The clinical difference between a simple fracture and combined injuries may be related to the different force transmission pathway. Recently, a systematic review of AMF fractures involving the analysis of 128 cases in 10 articles found that among 114 surgical patients, 80 cases (70.2%) underwent the LCL reconstruction (19).

### Medial collateral ligament complex

**Figure 4:**
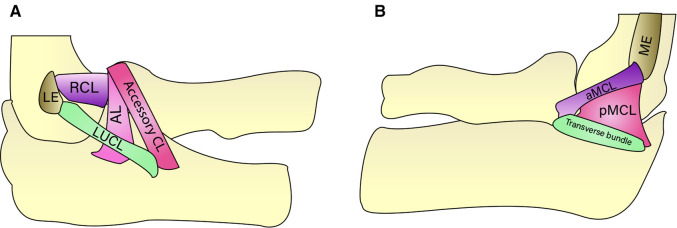
A lateral collateral ligament complex. Illustration showes the four components of the lateral collateral ligament complex: the annular ligament (AL), radial collateral ligament (RCL), lateral ulnar collateral ligament (LUCL), and the functionally irrelevant accessory collateral ligament (CL). 4B Medial collateral ligament. Illustration shows the medial collateral ligament components: the anterior (aMCL), posterior (pMCL), and the functionally irrelevant transverse bundles. LE: lateral epicondyle, ME: medial epicondyle.

A study focusing on the classification of elbow recurrent instability concluded that the injury or long-term overload was more likely to cause the rupture or chronic impairment of the MCL, and then induced the valgus instability of the elbow (21). With the violence transmission, valgus instability is most commonly in radial head fractures (R-M type 2 and O'Driscoll type 1) and then affects MCL. Controversially, some studies have shown that MCL injuries were common in simple radial head fractures, and the LCL injury was often involved in the combined patterns between coronoid and radial head fractures (15). Although previous studies have shown that AMFs were related to LCL rupture and rarely associated with MCL injury (11, 18), there was little literature showing that MCL injury occurs in O’Driscoll type 2. It is called upon orthopedists to consider potential MCL injury if the elbow stability was not restored after repair of the coronoid process and LCL, especially in the subtype 2 fracture type. O'Driscoll type 2–3 fracture involving sublime tubercle providing the attachment site for the anterior bundle of MCL was subjected to avulsion during elbow trauma (24). In total, coronoid fracture with MCL injury may be more complex than previously recognized (Figure 4).

## Treatment strategies based on classification

### Coronoid tip fracture

For the R-M type1 and O'Driscoll type1-1 coronoid tip fractures, the fragments were generally small and did not significantly affect the elbow stability (8), non-surgical treatment and close follow-up can be adopted (4, 5). Occasionally, ORIF was required when the presence of important ligament injuries or elbow instability (26–28).

Coronoid process with radial head fracture and elbow dislocation (TTIs) features a high incidence of elbow instability, arthritis, and stiffness (9). Based on these, most researchers recommend fracture fixation and scathing ligament repairing (5, 24, 25). However, certain studies reported favorable functional outcomes of TTIs after nonoperative treatment. Guitton (29) analyzed the non-surgical treatment of 4 TTIs featuring good elbow alignment, small fragments, slight displacement, and no significant movement limitation. Follow-up analysis (2–55 months) revealed that all elbows gained good alignment, and 3 had good outcomes based on MEPS, ROM, and Broberg-Morrey scores (30). Chan (31) performed non-operative treatment on 12 TTIs according to concentric joint reduction, and small fracture fragments. During the follow-up period of at least 12 months, all patients had satisfactory elbow ROM and clinical outcomes (average DASH score of 8 and MEPI of 94). Radiologically, no elbow instability was found. Therefore, non-surgical treatment combined with close follow-up can be used as an alternative to ORIF for TTIs meeting specific conditions.

**Figure 5:**
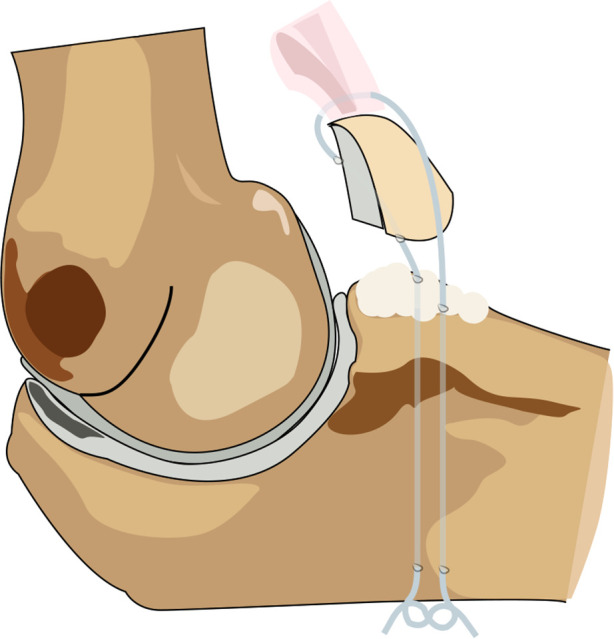
Suture lasso technique was used to fix the tip fracture of coronoid process.

The surgical management of TTIs was controversial. Garigues (25) retrospectively analyzed the fixator selection of 40 TTIs in 3 centers. The study compared the functional outcomes of 28 patients with suture lasso and 12 patients with screw or anchor fixations during a follow-up period of at least 18 months. Although there were no significant differences in MEPS, ROM, or DASH between the two groups, the stability of the coronoid process under lasso fixation was superior to that of the ORIF group after fixation, LUCL repair, and at the last follow-up. In addition, ORIF group was associated with a higher risk of implant failure. The study concluded that in TTIs with LUCL injuries, the suture lasso technique can be utilized to fix the coronoid fragments regardless of the classification (Figure 5).

Treatment approaches to radial head fractures can also impact the clinical outcome of the TTIs (32–34). A cohort study of 88 TTIs found that patients treated with radial head arthroplasty (RHA) had worse functional outcomes, smaller ROM, and more complications than patients treated with internal fixation of radial head fractures. Therefore, it was speculated that the treatment of radial head fracture could be an independent factor affecting the long-term outcome of TTIs (32). The minimized fixation of TTIs won satisfactory clinical results and wide ROM of the elbow. Furthermore, a retrospective study (33) of 14 TTIs (2 R-M type 1 and 12 type 2) showed that the MCL repair or external fixator could be considered instead of additional fixation for the coronoid if the elbow remains unstable after replacing or repairing the radial head and ligaments. In conclusion, for patients with TTIs, if complete repair and firm fixation of the radial head and ligament complex were achieved, additional coronoid internal fixation should be avoided to decrease postoperative complications such as joint stiffness and improve long-term functional outcomes (33, 34).

### AMF fractures

The integrity and stability of the coronoid anteromedial facet were the primary considerations during the clinical management of AMF fractures (7, 12, 13). Considering this fracture often results in early arthritis and elbow instability, most studies recommend internal fixation for AMFs. Surgical interventions of AMFs are controversial (24, 35, 36). Park (24) described the standard treatment for AMFs based on the acceptable clinical outcomes and elbow ROM after the management of 11 AMF fractures: LCL repairing alone for O'Driscoll type 2-1, fragments fixation with repairing LCL for O'Driscoll type 2-2, 2–3. The study highlighted the necessity of MCL injury detection if residual elbow instability after fragments fixation and collateral ligament repair. In addition, Rhyou (37) proposed another treatment strategy based on fragment size: surgery should be performed if fragments >6 mm, otherwise, conservative treatment or suture anchor fixation may be considered. Whether to perform fracture fixation alone, ligament repair alone, or a combination of both depends on the size of the fracture and the extent of soft tissue damage. Buttress plate fixation *via* a medial approach was also a reliable method of treatment that produces satisfactory and predictable outcomes (36).

However, some studies believe that even small fragments should be treated. Chen (20) treated 20 AMF fractures: screw fixation for large, reducible, and non-comminuted fractures; small osteosynthesis plate or coronoid process plate for cleavage fractures on the coronoid anteromedial edge; screw and suture anchors for the cases of avulsion fracture with sublime tubercle. After 2 years of follow-up, 18 patients (90%) had good clinical outcomes, with an average MEPS of 88 and quick-DASH of 7.

Some studies also reported that conservative treatment in specific AMF fractures can achieve an acceptable functional outcome (38, 39). Moon (38) nonoperatively treated 3 AMF fractures (2 O'Driscoll type 2–3 and 1 type 2–3), carried out a mean follow-up of 2 years, and found that 3 patients, both in clinical evaluation and radiographic findings, obtained complete ROM, excellent MEPS scores, and elbow instability in the varus stress test. Chan (39) non-surgically treated 10 AMF fractures (9 type 2–3 and 1 type 2–3) and conducted an average follow-up of 50 months. Patients had mean MEPS of 94 (6 excellent and 4 good), DASH of 7, and ROM flexion of 137 degrees. Functional scores, ROM, and muscle strength of the upper extremity were not significantly different from those of the contralateral forearm. Non-surgical treatment has strict indications involving non-displaced or slight displaced fractures, good alignment of the elbow, and no elbow instability under varus stress test. However, the evaluation of these specific situations is subjective and lacks quantitative research.

### Basal fracture

Coronoid basal fracture, R-M type 3 and O'Driscoll type 3 fracture, features olecranon fracture-dislocation and few soft tissue injuries (11, 14, 15). In coronoid basal fracture with anterior olecranon fracture-dislocation, the fracture fragment is usually large, single, and of good bone quality. Safe and effective internal fixation can be achieved with screws or plates. However, with posterior olecranon fracture-dislocation, the basal fracture is of varying sizes and is often associated with radial head fracture and LCL injury making surgery more complex. The support plates can deal with fractures with large fragments and good bone mass, while the small transverse fracture with joint capsule insertion can be fixed by suture lasso. If combined with osteoporosis or complex injuries, it is necessary to strengthen the fixation with hinge support or interlocking nail external fixation. Coronoid process reconstruction or prosthetic replacement is available when excessive bone loss or irreducible comminuted fragments (2, 26, 40).

### Treatment principles

**Figure 6:**
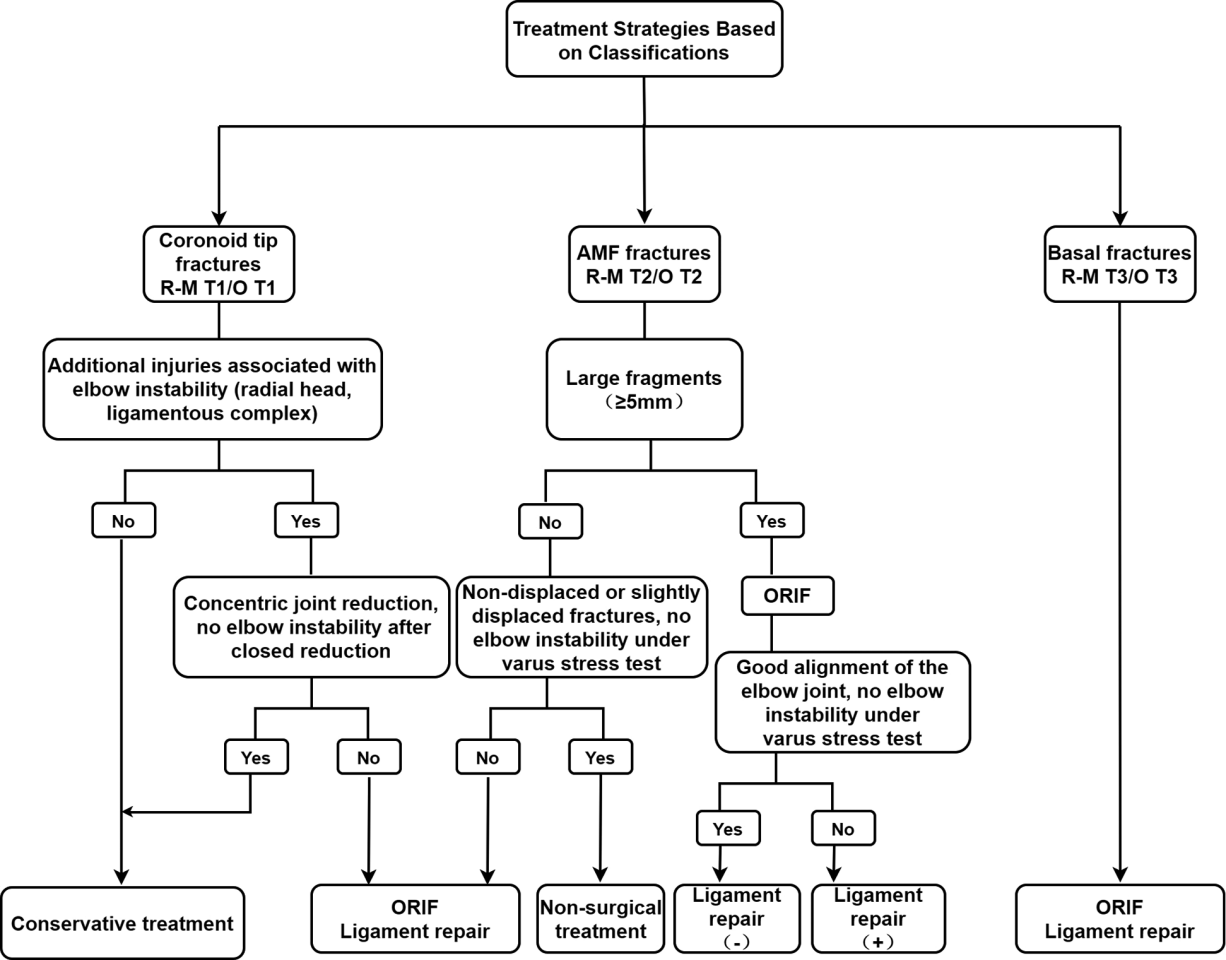
Treatment strategies based on classifications.

The prior consideration in the clinical management of coronoid fractures is to restore and maintain elbow stability (4, 5, 7, 13, 26). Non-operative treatment may be considered when non-displaced or slightly displaced fracture, small fragments and negative lateral stress test under fluoroscopy. Surgical intervention is designed to restore the good alignment of the proximal ulnar trochlear notch and to reduce the unstable elbow while repairing the ligament and managing the combined injury (7, 26) (Figure 6).

## Area of future research

Current coronoid fracture classifications were certainly proposed based on the fragment features and injury pattern. It is uncertain what exactly mechanism and associated injuries correspond to the classification. Research in this orientation is extremely important because of its relevance to clinical management. Preliminary data from fracture line mapping have indicated partial mechanisms of elbow trauma and proposed a new coronoid classification (14, 15), but the clinical significance of the new type changes is unclear. Further studies are needed to assess the correlations between associated injuries and coronoid fracture. More patient data are needed.

## Summary

Coronoid process plays an important role in maintaining the stability of the elbow and is easily subjected to fracture in elbow trauma. The classification of coronoid fracture can help to predict fracture patterns, guide treatment, and improve prognosis. However, with the in-depth study and understanding of the coronoid fracture, we found there are still many controversies regarding the fracture location and treatment of the existing classification system. In addition, the existing classification does not include the combined injuries information that may explain the elbow traumatic mechanism, stability, and surgical management. Therefore, the present coronoid fracture classification systems still need to be further optimized.
